# Hoarding knowledge or hoarding stress? Investigating the link between digital hoarding and cognitive failures among Chinese college students

**DOI:** 10.3389/fpsyg.2024.1518860

**Published:** 2025-01-30

**Authors:** Yan Liu, Yaorong Liu

**Affiliations:** ^1^School of Media and Communication, Shenzhen University, Shenzhen, China; ^2^School of Communication and Media, Guangzhou Huashang College, Guangzhou, China

**Keywords:** digital hoarding, fatigue, mindfulness, cognitive failures, digital information management

## Abstract

**Introduction:**

Digital hoarding is defined as the persistent accumulation of digital content and an unwillingness to delete it. This behavior has been found to be particularly prevalent among young people. This study aims to explore the impact of digital hoarding on cognitive failures, examining fatigue as a mediator and mindfulness as a moderator.

**Method:**

A total of 801 participants were recruited to complete a survey that contained measures of digital hoarding, fatigue, mindfulness, and cognitive failures. The hypothesized moderated mediation model was tested using Models 4 and 8 from the PROCESS macro in SPSS.

**Results:**

(1) Digital hoarding positively predicted cognitive failures. (2) Fatigue mediated the relationship between digital hoarding and cognitive failures. (3) Mindfulness moderated both the direct effect of digital hoarding on cognitive failures and the first segment of the mediating role of fatigue.

**Discussion:**

This study contributes to a deeper understanding of digital hoarding. It also highlights the great potential of mindfulness in mitigating the negative effects of digital hoarding, and provides students with practical strategies for developing healthier and more balanced digital habits.

## 1 Introduction

With the rapid development of information technology, the Internet has significantly changed the way people access information and consume media (Tandoc Jr et al., 2019). Social media and cloud services allow users to download, share and store various digital re-sources without the limitations of physical storage (Sillence et al., 2023a). However, despite the convenience, this ease of access has created challenges, such as the phenomenon of “digital hoarding.” Digital hoarding refers to the persistent accumulation of digital content and the reluctance to delete it (Sweeten et al., 2018). Data shows that millions of Americans have inboxes with more than 1,000 unread emails, and 60% of Americans never delete pictures or videos from their digital devices (Ghlionn, 2023). The report suggests that the prevalence of pathological digital hoarding in non-clinical samples ranges from 3.7 to 6% in the general population, with a significantly higher rate of 21.5% observed in younger individuals (Bulli et al., 2014). This indicates that as everyday life becomes increasingly digital, digital hoarding is becoming more common as a distinct form of behavioral disorder (Thorpe et al., 2019). Although scholars have examined digital hoarding from various perspectives and provided valuable insights, certain gaps in the research remain.

First, as an emerging concept, research on digital hoarding behavior is still in its early stages (Neave et al., 2019; Vinoi et al., 2024). Current academic discussions are largely grounded in theoretical analysis and qualitative interviews (Luxon et al., 2019; McKellar et al., 2020; McKellar et al., 2024), while empirical research in this field is comparatively much less. The existing literature does not fully reveal the causes and consequences of digital hoarding behavior.

Second, the limited quantitative research available primarily focuses on the antecedents of digital hoarding (Agarwal et al., 2024; Sedera et al., 2022; Liu et al., 2024), while studies on its adverse consequences are notably scarce. Investigating the negative effects of this behavior is crucial, particularly for promoting responsible social media use and improving digital information management among students. A recent exploratory study identified a significant negative correlation between digital hoarding and cognitive failures (Lian et al., 2023); however, this finding lacks sufficient empirical evidence. More importantly, the mechanisms underlying the relationship between digital hoarding and cognitive failures—specifically how and when this impact occurs—remain unclear and warrant further investigation.

Third, existing research seldom isolates college students from the general population for focused study. However, studies suggest that digital hoarding is more prevalent among college students today (Wang et al., 2022), highlighting the need for more targeted re-search on this group. Erich Fromm argued that contemporary education systems pro-mote a “having-oriented” approach to knowledge acquisition, where knowledge is viewed as a means of enhancing self-worth and securing future social status (Fromm, 1976). This is particularly evident in the current social context, where China’s economic downturn and rising unemployment have intensified social competition and division. To increase their future competitiveness and sense of job security, college students are increasingly engaging in digital hoarding, accumulating vast amounts of digital information (Liu et al., 2024). In other words, confronted with academic and employment pressures, they tend to view accumulation as a means of securing a competitive edge and keeping up with their peers, rather than prioritizing the immediate utility of the content. This behavior not only fosters digital hoarding and exacerbates disorganized information management, but may also trap individuals in a vicious cycle of mental fatigue and physical exhaustion, negatively impacting cognitive function and emotional regulation.

Based on this, the current study aims to construct a moderated mediation model to explore the relationship between digital hoarding behavior and cognitive failures among college students. It also examines the mediating role of fatigue and the moderating effect of mindfulness. This research has important theoretical and practical implications for improving the efficiency of digital information management, reducing cognitive stress and promoting the mental health of students.

## 2 Literature review and research hypothesis

### 2.1 Digital hoarding and cognitive failures

Individuals with hoarding tendencies often experience significant functional impairments and a decline in overall quality of life (Saxena et al., 2011). Cognitive failures are cognition-based slips and lapses in simple tasks that a person can usually perform without errors (Lange and Süß, 2014), including attention, memory, and motor failures (Wallace et al., 2002). According to the theory of attentional overload, an individual’s attentional resources are limited. When multiple cognitive functions are activated simultaneously, excessive consumption of mental resources can occur, increasing the likelihood of cognitive failures (Head and Helton, 2014). Previous research has shown that digital files stored with identical icons and in close spatial proximity can burden working memory, consume more cognitive control resources and impair people’s ability to selectively focus attention (Gil-Gómez de Liaño et al., 2016). Boardman and Sass also pointed out that when faced with an overwhelming amount of resources, people tend to hesitate to categorize and prioritize files that have emotional value or require deep processing. This indecision draws their attention to digital items, making it difficult to focus on other tasks and leading to cognitive overload and emotional fluctuations (Boardman and Sasse, 2004).

In addition, according to the “Google effect” in memory (Sparrow et al., 2011), digital hoarders tend to over-rely on digital devices to store knowledge, treating them as memory partners. As a result, individuals remember the location of the information rather than the content itself, making it difficult to perform even simple cognitive tasks (Gong and Yang, 2024). A recent study also found a significant positive correlation between knowledge hoarding and cognitive failures (Lian et al., 2023). Therefore, digital hoarding as a major source of stress may negatively affect cognitive processes and behavioral outcomes. Based on this, we made the following hypothesis:

*Hypothesis* 1: Digital hoarding can positively predict cognitive failures.

### 2.2 Fatigue as a mediator

Fatigue refers to the physical and psychological exhaustion perceived by an individual (Michielsen et al., 2003). Research has shown that negative emotions, such as fatigue and burnout, are significant contributors to cognitive failures (Hu et al., 2017). Prolonged fatigue not only impairs cognitive processing efficiency, but also negatively affects key cognitive functions such as attention, memory and decision-making (Van Gaeveren et al., 2024). The Conservation of Resources (COR) theory suggests that the more negative emotions an individual accumulates, the more depleted their cognitive resources become (Hobfoll, 2001). Consequently, they struggle to allocate cognitive resources effectively and consistently when completing tasks, leading to increased cognitive failures (Yaakoubi et al., 2024). Yu et al. also found that fatigue directly predicts cognitive failures among college students (He et al., 2022).

Moreover, digital hoarding may contribute to feelings of fatigue. Uncontrolled downloading over long periods of time, or habitual “one-click saving,” exposes individuals to an overwhelming amount of digital resources (Sillence et al., 2023b). According to the individual-environment fit model of stress, when an individual’s cognitive abilities do not match the demands of a highly stressful, complex information environment, they become overwhelmed, leading to a state of “struggling to cope” and resulting in both physical and mental exhaustion (Andreassen, 2015). Related studies also suggest that an individual’s level of hoarding can significantly predict their level of anxiety and depression (Tolin et al., 2018; Raines et al., 2016). Therefore, fatigue may serve as a mediating variable, bridging the relationship between digital hoarding and cognitive failures. Based on the above reasoning, we proposed the following hypothesis:

*Hypothesis* 2: Fatigue mediates the relationship between digital hoarding and cognitive failures.

*Hypothesis* 2a: Fatigue can positively predict cognitive failures.

*Hypothesis* 2b: Digital hoarding can positively predict fatigue.

### 2.3 Mindfulness as a moderator

The Individual-Environment Interaction Theory posits that an individual’s psychology and behavior are shaped by interactions between the individual and his or her environment (Lerner, 2004). This implies that the effect of digital hoarding on cognitive processes and behavioral outcomes may be moderated by individual characteristics.

Mindfulness involves being aware of the present moment in an open and non-judgmental way (Baer, 2003). Numerous studies have demonstrated the protective role of mindfulness in countering problematic online behavior (Giancola et al., 2024). For example, the study by Meynadier et al. (2024) found that lower levels of mindfulness were strongly associated with more problematic social media use. A recent exploratory study also found that mindfulness negatively predicted digital hoarding behavior among young people (Liu et al., 2024).

Mindfulness is also an important safeguard for mental health (Tran et al., 2024). Meta-analyses have shown that mindfulness can effectively reduce negative emotions and improve overall well-being (Zhang et al., 2021). Liu et al. (2020) found that loving-kindness meditation (LKM), a new subspecies of mindfulness, has the effect of generating positive emotions and fostering positive attitudes (Chiou et al., 2022; Chen et al., 2023). Previous research suggests that digital hoarding is essentially the result of a combination of negative emotions, such as fear of missing out and loss aversion, and is closely linked to the depletion of self-control resources (Vinoi et al., 2024; Wu et al., 2024). This implies that compared to digital hoarders with lower mindfulness, those with higher mindfulness are better equipped to manage their negative emotions, potentially reducing the depletion of self-control resources. This may help to prevent the onset of fatigue and cognitive failure to some extent. Based on the above reasoning, we hypothesized the following:

*Hypothesis* 3: Mindfulness moderates the relationships between digital hoarding, fatigue and cognitive failures.

*Hypothesis* 3a: The direct effect of digital hoarding on cognitive failures will be moderated by mindfulness.

*Hypothesis* 3b: The first half of the mediation effect, namely the influence of digital hoarding on fatigue, will be moderated by mindfulness.

### 2.4 The present study

**Figure 1:**
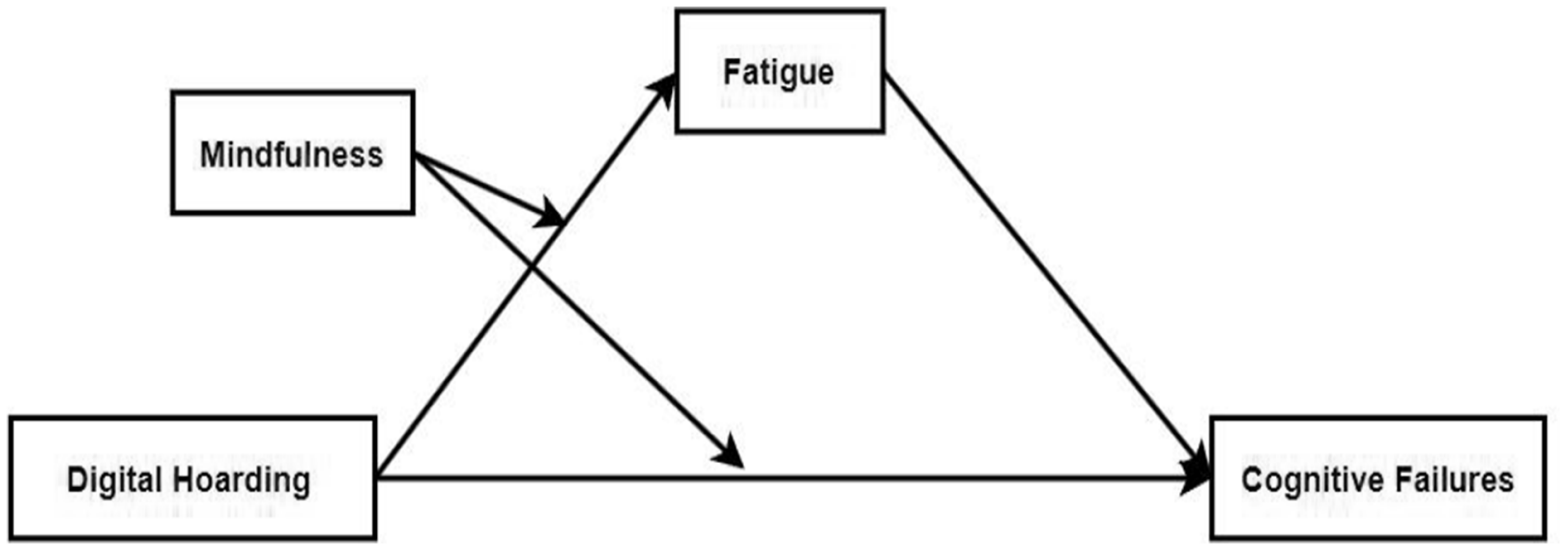
Hypothesized moderated mediation model.

Taken together, this study constructs a moderated mediation model. The aims of the current study are threefold. First, it examines the relationship between digital hoarding and cognitive failures. Second, it tests whether fatigue mediates the relationship between digital hoarding and cognitive failures. Third, it explores whether mindfulness moderates both the direct and indirect relationships between digital hoarding and cognitive failures. The moderated mediation model is presented in Figure 1.

## 3 Methods

### 3.1 Participants

The survey was conducted over a three-month period, from 10 June to 19 September 2024. This study employed convenience and random sampling methods and recruited a total of 801 Chinese university students to participate in the survey. The sample consisted of 439 males (54.8%) and 362 females (45.2%) aged between 18 and 25 years (*M* = 19.44, SD = 1.33). The participants included 168 freshmen, 246 sophomores, 261 juniors and 126 seniors.

### 3.2 Measures

#### 3.2.1 Digital hoarding

Neave et al. developed the Digital Behaviors Questionnaire (DBQ) to measure digital hoarding behavior (Neave et al., 2019). While this scale is effective in assessing individuals’ digital hoarding tendencies, it has certain limitations, such as focusing primarily on email hoarding in the workplace and overemphasizing emotional factors. To address these issues, Wu et al. revised the scale to better fit the social media context (Wu et al., 2024). The revised scale consists of 7 items and includes two dimensions: “accumulation” and “difficulty in discarding.” The “accumulation” dimension consists of 4 items, reflecting the external behavioral characteristics of individuals who continuously hoard digital files, such as “I believe some social media files may be useful in the future.” The “difficulty in discarding” dimension contains 3 items, indicating difficulty in deleting digital files due to learning or emotional reasons, such as “I find it hard to delete unused social media materials.” Participants rate each item on a scale from 1 (strongly disagree) to 5 (strongly agree), with no reverse scoring. The total score is the sum of all items, with higher scores indicating greater levels of digital hoarding. This scale is commonly used to assess digital hoarding behaviors among Chinese youth and has demonstrated good reliability and validity (Liu et al., 2024). In this study, the Cronbach’s *α* for the scale was 0.81.

#### 3.2.2 Fatigue assessment scale

The Fatigue Assessment Scale, developed by Michielsen et al. and revised by Chen et al., was used in this study (Kerr and Levine, 2008). This scale has been adapted for different populations and the Chinese version has demonstrated high reliability and validity in assessing levels of fatigue (Chen et al., 2019). The Fatigue Scale contains 10 items, with representative items such as “I feel somewhat exhausted,” “I find it difficult to start tasks,” and “My thoughts are sometimes unclear.” Participants rate each item on a 5-point scale from 1 (never) to 5 (always). The average score across all items is calculated, with higher scores indicating greater perceived fatigue. In this study, the Cronbach’s *α* coefficient for the scale was 0.88.

#### 3.2.3 Cognitive failure questionnaire

The Cognitive Failure Questionnaire (CFQ), originally developed by Broadbent et al. and revised by Zhang et al., was used in this study (Broadbent et al., 1982; Zhang, 2013). The CFQ includes three dimensions: attention failures, memory failures, and action execution failures, with a total of 18 items. Typical items include “I often forget where I put things” and “I get easily distracted while reading,” The questionnaire uses a 5-point Likert scale (1 = “strongly disagree,” 5 = “strongly agree”). The total score is calculated by summing the scores for each item, with a higher score indicating a higher frequency of cognitive failures. This scale has been demonstrated to be highly valid and reliable in Chinese undergraduate students and clinical samples (Zhang et al., 2020). In this study, the Cronbach’s *α* for the scale was 0.91.

#### 3.2.4 Mindfulness

The Child and Adolescent Mindfulness Measure (CAMM), developed by Greco et al. and revised into Chinese by Liu et al., was used to assess mindfulness (Greco et al., 2011; Liu et al., 2019). This scale consists of 10 items, such as “I have trouble focusing on one thing” and “I often think about the past rather than the present” The items are rated on a 5-point scale, where 0 indicates “never” and 4 indicates “always.” All items are reverse-scored, and the average score is calculated, with higher scores indicating higher levels of mindfulness in daily life. This scale is widely used with adolescents and college students, and it has shown good reliability and validity (Dion et al., 2018; Yang et al., 2021). In this study, the Cronbach’s α for the scale was 0.89.

### 3.3 Procedure

Before the formal study, we conducted a pilot test with 107 active digital hoarders. The results indicated that the questionnaire had an acceptable level of reliability and validity.

During the formal research phase, we used the Wenjuanxing platform^1^ to manage and distribute the questionnaires. Participants were recruited through both online and offline channels. Specifically, we posted the survey link on four major Chinese social media platforms: Weibo, WeChat, Xiaohongshu, and Zhihu. As mainstream social media platforms in China, they share several common characteristics (Liu et al., 2024): (1) a diverse range of content, including images, short videos, professional articles, and user-shared life experiences; (2) an emphasis on user interaction, allowing users to build social networks while keeping up to date with the digital activities of others; (3) algorithmic systems that curate content based on users’ interests and social connections; (4) most importantly, these platforms offer bookmarking functions for content collection and host interest groups focused on digital hoarding, which enabled us to obtain more targeted and representative samples. In addition, as a complementary method, we distributed the questionnaire by posting QR codes in offline locations to reach a wider target population. We selected different locations such as campuses, libraries and restaurants - public spaces frequented by university students - to increase the diversity and representativeness of the sample. This approach aimed to minimize the bias of relying on a single sample source and to ensure that the study results would be more generalizable and applicable.

In order to accurately identify participants, we referred to previous research and included screening questions at the beginning of the questionnaire (Wu et al., 2024). Screening questions included: “Are you a student?,” “Are you currently an active user of social media platforms?,” and “Have you bookmarked any social media content in the past two weeks?.” Participants who answered “no” to any of these questions were excluded from the survey. Additional criteria for filtering valid samples included excessively short response times (less than 3 min) or consistent answers throughout. We also included attention check questions (e.g., “What is the capital of China?”) to ensure participant engagement. This study was approved by the ethics committee of the first author’s university. Before the survey began, all participants signed an online informed consent form and were informed that they could refuse or withdraw from the study at any time. After completing the questionnaire, participants received a reward of 1 to 3 RMB.

### 3.4 Statistical analysis

Data were processed using SPSS 26.0 and Process 3.3. First, descriptive statistics were computed, and Pearson correlations were conducted to examine the relationships among digital hoarding, fatigue, mindfulness, and cognitive failures. Second, Hayes’ SPSS PROCESS macro (Model 4 and Model 8) was used to examine the mediating role of fatigue and the moderating role of mindfulness (Hayes, 2013). All regression coefficients were tested using the bias-corrected percentile Bootstrap method, and the 95% confidence interval (CI) was calculated through 5,000 resampled iterations. All variables were standardized prior to formal data analysis.

## 4 Results

### 4.1 Common method biases

Self-reported data may introduce common method bias. To enhance the scientific rigor of this study, Harman’s single-factor test was conducted to assess this bias. The results revealed seven factors with eigenvalues greater than 1, with the first factor accounting for only 30.91% of the total variance, which is below the critical threshold of 40% (Hair et al., 2014). These results suggest that common method bias is not a significant concern in this study.

### 4.2 Correlation analysis

All variables were normally distributed, as the skewness and kurtosis values fell within acceptable ranges (skewness < |2.0| and kurtosis < |7.0|; Hancock et al., 2018; Kline, 2023; Byrne, 2016). The independence of residuals was confirmed (1 < D-W < 2), and no multicollinearity was detected (VIF < 5; Hair et al., 2014; Byrne, 2016). This means that the data is suitable for further analysis.

Table 1 presents the means, standard deviations, and correlation coefficients for the variables. The results showed significant correlations between digital hoarding, fatigue, cognitive failures, and mindfulness. Specifically, digital hoarding was positively correlated with fatigue (*r* = 0.37, *p* < 0.01) and cognitive failures (*r* = 0.36, *p* < 0.01). Fatigue was positively correlated with cognitive failures (*r* = 0.47, *p* < 0.01). Mindfulness was significantly negatively correlated with digital hoarding (*r* = −0.48, *p* < 0.01), fatigue (*r* = −0.44, *p* < 0.01), and cognitive failures (*r* = −0.36, *p* < 0.01).

**Table 1:** Descriptive statistics and correlations for the main study variables.

	M	SD	1	2	3	4
1.Digital hoarding	3.54	0.73	1			
2.Fatigue	3.30	0.75	0.37**	1		
3.Cognitive failures	3.14	0.81	0.36**	0.47**	1	
4.Mindfulness	1.77	0.76	−0.48**	−0.44**	−0.36**	1

**p < 0.001.

### 4.3 Testing the mediation effect

First, using Model 4 (a simple mediation model) from the PROCESS macro in SPSS, we applied 5,000 bootstrap resamples to the original sample. Controlling for variables such as gender, age, and grade, we examined the mediating effect of fatigue in the relationship between digital hoarding and cognitive failures.

The results of the regression analysis (see Table 2) indicated that digital hoarding significantly predicted cognitive failures (*β* = 0.36, *p* < 0.001). Even after including the mediating variable, the direct effect of digital hoarding on cognitive failures remained significant (*β* = 0.21, *p* < 0.001). Additionally, digital hoarding significantly predicted fatigue (*β* = 0.38, *p* < 0.001), and fatigue significantly predicted cognitive failures (*β* = 0.39, *p* < 0.001). Hypotheses 2a and 2b were supported.

**Table 2:** Testing the mediation model.

Predictors	Model 1 Cognitive failures		Model 2 Fatigue		Model 3 Cognitive failures	
	*B*	*t*	*B*	*t*	*B*	*t*
Gender	−0.03	−1.08	−0.004	−0.08	−0.03	−0.68
Age	−0.01	−0.12	0.01	0.12	0.04	1.03
Grade	0.01	0.27	−0.01	−0.40	0.01	0.27
Digital hoarding	0.36	10.81***	0.38	11.31***	0.21	6.37***
Fatigue					0.39	12.29***
*R2*	0.13		0.14		0.27	
*F*	30.26***		32.02***		58.96***	

***p < 0.001.

Furthermore, the bootstrap 95% confidence intervals for both the direct effect of digital hoarding on cognitive failures and the mediating effect of fatigue did not contain 0 (see Table 3), indicating that digital hoarding not only directly predicts cognitive failures but also indirectly leads to cognitive failures through the mediating role of fatigue. The direct effect (0.21) and the mediating effect (0.15) accounted for 58.3 and 41.7% of the total effect (0.36), respectively. Hypothesis 2 was confirmed.

**Table 3:** Decomposition of total, direct and indirect impacts.

Effect type	*B*	BootSE	BootLLCI	BootULCI
Total effect	0.36	0.03	0.29	0.43
Direct effect	0.21	0.03	0.15	0.27
Indirect effect	0.15	0.02	0.11	0.19

### 4.4 Testing the moderated mediation model

Next, Hayes (2013) SPSS macro Model 8, which assumes that both the first half of the mediation model and the direct pathway are moderated (consistent with the theoretical model of this study), was used to test the moderated mediation model. The analysis was conducted while controlling for gender, age, and grade level to ensure the robustness of the findings.

The results (see Table 4) show that, after introducing mindfulness into the model, digital hoarding significantly positively predicted fatigue (*β* = 0.34, *p* < 0.001), fatigue significantly positively predicted cognitive failures (*β* = 0.32, *p* < 0.001), and digital hoarding significantly positively predicted cognitive failures (*β* = 0.25, *p* < 0.001).

**Table 4:** Testing the moderated mediation model.

Predictors	Model 1 Fatigue		Model 2 Cognitive failures	
	*B*	*t*	*B*	*t*
Gender	−0.01	−0.19	−0.03	−0.61
Age	−0.002	−0.04	0.04	1.05
Grade	−0.02	−0.66	−0.06	−1.78
Digital Hoarding	0.34***	8.61	0.25***	6.15
Mindfulness	−0.33***	−9.61	−0.12***	−3.58
Fatigue			0.32***	9.31
Digital Hoarding x Mindfulness	−0.28***	−7.35	−0.16***	−4.26
*R2*	0.28		0.30	
*F*	50.83		47.82	

***p < 0.001.

Meanwhile, the interaction between digital hoarding and mindfulness showed a significant negative predictive effect on both cognitive failures (*β* = −0.16, *p* < 0.001) and fatigue (*β* = −0.28, *p* < 0.001). This indicates that mindfulness moderates not only the direct relationship between digital hoarding and cognitive failures but also the relationship between digital hoarding and fatigue.

To better illustrate the moderating effect, simple slope plots were generated at different levels of mindfulness.

**Figure 2:**
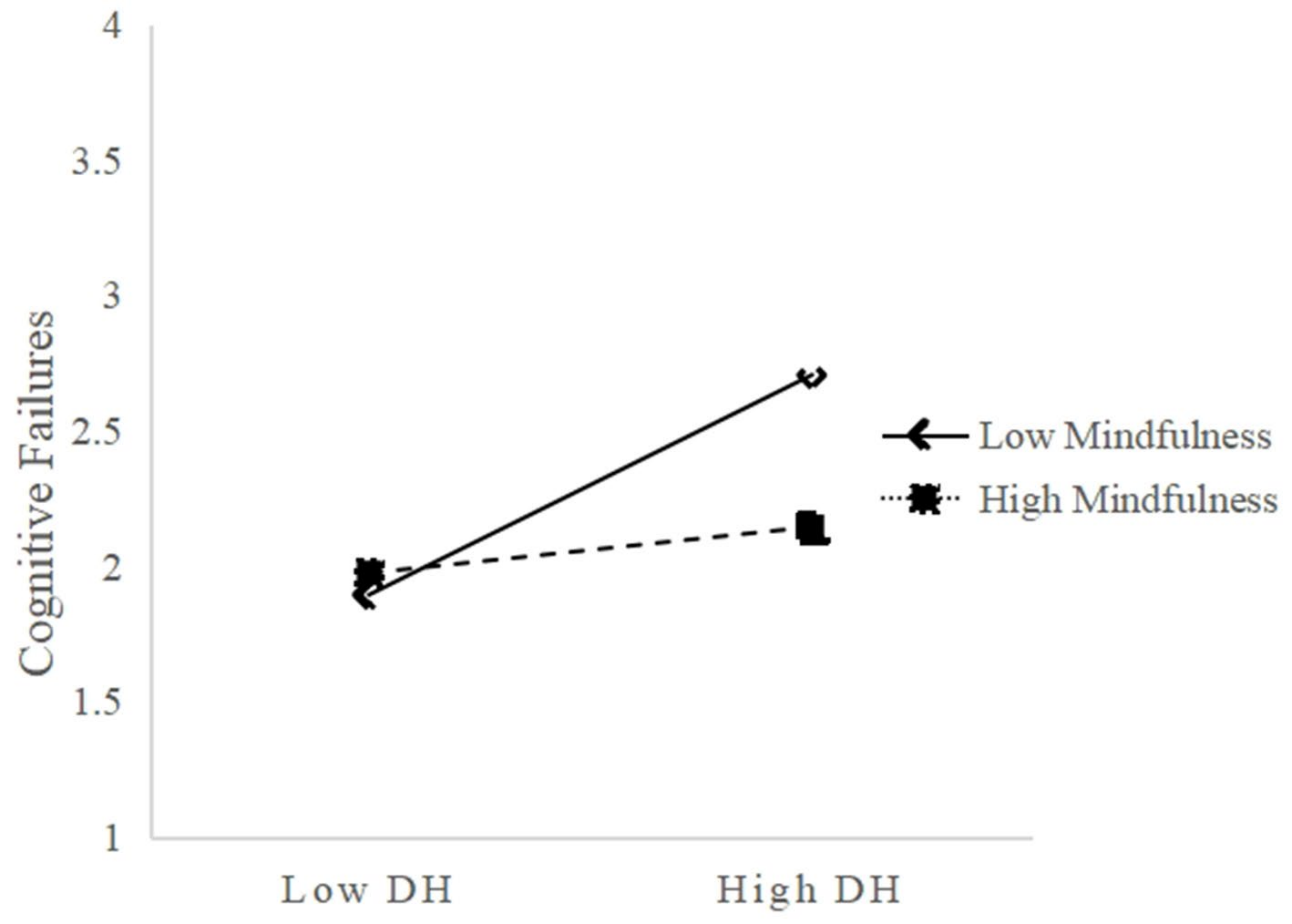
Mindfulness moderates the relationship between digital hoarding and cognitive failures. DH, Digital Hoarding.

As shown in Figure 2, for participants with lower mindfulness levels (M-1SD), digital hoarding had a significant positive predictive effect on cognitive failures (*β* = 0.37, *p* < 0.001). For participants with higher mindfulness levels (M + 1SD), while digital hoarding still positively predicted cognitive failures, the effect was much smaller (*β* = 0.12, *p* < 0.001). This suggests that as an individual’s level of mindfulness increases, the predictive effect of digital hoarding on cognitive failures gradually decreases (see Table 5). Hypothesis 3a was therefore supported.

**Table 5:** Conditional direct effect of digital hoarding on cognitive failures.

Mindfulness	Effect	BootSE	BootLLCI	BootULCI
M-1SD	0.37	0.06	0.25	0.49
M	0.25	0.04	0.17	0.32
M + 1SD	0.12	0.04	0.05	0.19

**Figure 3:**
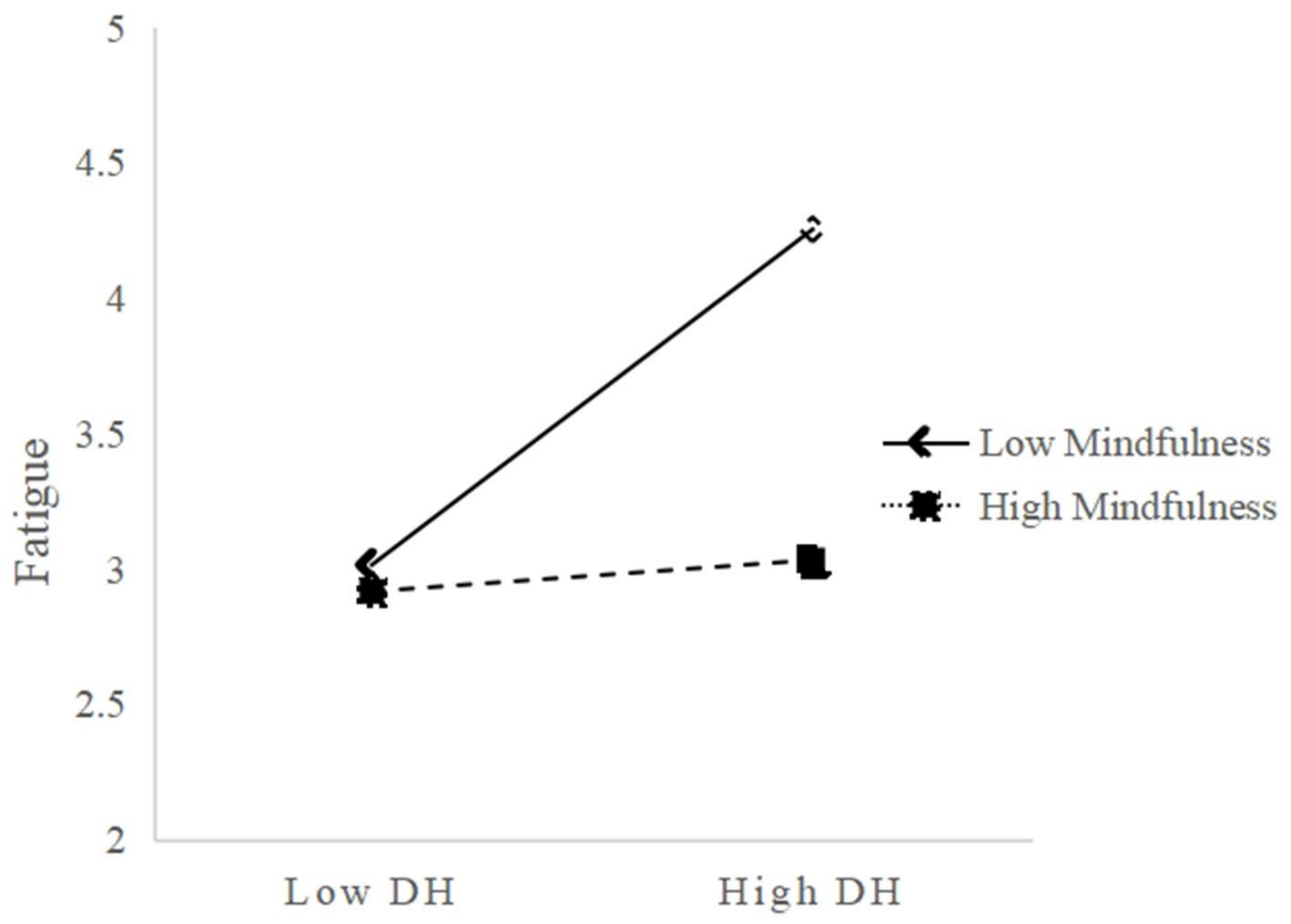
Mindfulness moderates the relationship between digital hoarding and fatigue. DH, Digital Hoarding.

As shown in Figure 3, for participants with lower mindfulness levels (M-1SD), digital hoarding significantly predicted fatigue (*β* = 0.55, *p* < 0.001). For participants with higher mindfulness levels (M + 1SD), digital hoarding still positively predicted fatigue, but the effect was notably reduced (*β* = 0.13, *p* < 0.001). This demonstrates that as an individual’s mindfulness increases, the predictive effect of digital hoarding on fatigue gradually diminishes. Therefore, Hypothesis 3b was supported.

In addition, at the three levels of mindfulness, the mediating effect of fatigue in the relationship between digital hoarding and cognitive failures, along with its 95% confidence intervals, was as follows: 0.18 [0.12, 0.24], 0.11 [0.07, 0.15], and 0.04 [0.01, 0.07]. This indicates that as mindfulness increases, the mediating effect of fatigue in the relationship between digital hoarding and cognitive failures gradually weakens (see Table 6). And up to this point, Hypothesis 3 was confirmed.

**Table 6:** Conditional indirect effect of digital hoarding on cognitive failures.

Mindfulness	Effect	BootSE	BootLLCI	BootULCI
M-1SD	0.18	0.03	0.12	0.24
M	0.11	0.02	0.07	0.15
M + 1SD	0.04	0.02	0.01	0.07

## 5 Discussion

This study constructed a moderated mediation model to explore the impact of digital hoarding on cognitive failures among college students and its underlying mechanisms. The empirical results showed that digital hoarding not only directly leads to cognitive failures but also indirectly influences cognitive failures through the fatigue pathway. Furthermore, mindfulness moderated both the direct and indirect effects of digital hoarding on cognitive failures.

### 5.1 The mediating role of fatigue

This study is the first to empirically confirm the critical role of fatigue in the relationship between digital hoarding and cognitive failures, with the mediating effect accounting for almost half of the total effect. This means that individuals with higher levels of hoarding experience stronger feelings of fatigue and, consequently, more cognitive errors. This can be explained from two perspectives:

First, numerous studies have demonstrated that social media fosters upward social comparison, where users evaluate themselves against others who seem more successful or happier, often resulting in feelings of inadequacy and pressure (Chou and Edge, 2012; Liu Y, et al., 2022). Consequently, digital hoarders may develop a cognitive bias while gathering information on social media, believing that others are more accomplished and possess greater digital resources. This distorted perception exacerbates feelings of inferiority, prompting digital hoarders to compulsively bookmark resources, which perpetuates emotional resource depletion and may eventually lead to physical and mental exhaustion. These findings are further supported by the Conservation of Resources (COR) theory (Hobfoll, 2001), which suggests that an individual’s attentional resources are finite and that the accumulation of excessive digital items depletes these resources.

Second, the study also reveals that fatigue is a key antecedent variable that triggers cognitive failures. This finding aligns with the Cognitive Failure Integration Model, which suggests that excessive fatigue leads to a decline in cognitive functions such as working memory, executive function and attention. This impairment makes it difficult for individuals to consistently and effectively allocate their available cognitive resources during task execution and situational activities, resulting in frequent cognitive failures (Li et al., 2024). Spiegelberg et al. (2021) also demonstrated that higher levels of fatigue can cause thinking to become rigid and sluggish, significantly increasing the likelihood of cognitive failures. Therefore, fatigue may impair the ability of digital hoarders to perform everyday tasks effectively.

### 5.2 The moderating role of mindfulness

The study found that the effect of digital hoarding on cognitive failures was moderated by mindfulness. This finding is supported by the Individual-Environment Interaction Theory and suggests that mindfulness, as a positive individual trait, can act as a buffer against the effects of digital hoarding on emotional and cognitive adjustment. We can explain this from the following perspectives.

First, this finding is consistent with the Distraction-Conflict Theory (DCT), which suggests that mindfulness can improve cognitive performance by strengthening self-regulation and reducing “attentional conflict” caused by external stimuli (Jankowski and Holas, 2020). As a result, compared to low mindfulness individuals, high mindfulness individuals are better able to maintain attention and consciously redirect attention away from irrelevant stimuli. This allows them to stay focused and engaged in tasks, reducing the likelihood of mental distractions or “mind-wandering,” and ultimately resulting in fewer cognitive failures.

Second, the limited self-control theory posits that sufficient psychological resources are essential for effective self-control (Baumeister, 2002). Digital content overload depletes a significant amount of these resources, undermining self-control and contributing to cognitive dysfunction. Mindfulness, however, can replenish self-control resources and enhance an individual’s capacity for self-regulation. Furthermore, from a positive psychology perspective, mindfulness serves as an effective tool for fostering positive psychological states (Liu C, et al., 2022) and significantly increases physiological arousal levels (Broderick and Jennings, 2012). Therefore, for digital hoarders with high levels of mindfulness, even if they experience physical or mental fatigue during excessive accumulation of digital items, their stronger emotional regulation skills allow them to avoid depleting too much self-control and maintain cognitive effectiveness. This finding suggests that mindfulness not only alleviates feelings of fatigue, but also helps individuals break out of the vicious cycle of “irrational hoarding - fatigue - cognitive dysfunction.”

## 6 Implications

### 6.1 Theoretical implications

First, unlike previous studies that treated digital hoarding as an outcome variable, this study positions digital hoarding behavior as an independent variable and focuses on the negative consequences of this irrational hoarding. Through empirical analysis, the study validates inferences from prior qualitative research, thereby enriching the understanding and knowledge of digital hoarding as an emerging concept. In other words, the findings of this study make a valuable contribution to the existing academic discourse on digital hoarding by highlighting the link between this problematic behavior and cognitive failures in college students.

Second, this study further explores the mediating role of fatigue through the lens of Conservation of Resources (COR) theory, offering a novel explanation of the mechanisms by which digital hoarding leads to cognitive failures. It suggests that the negative impact of digital hoarding on cognitive and behavioral adaptation primarily operates through individuals’ internal experiences, such as fatigue. This theoretical insight helps to clarify how “digital possession” in the information age can impair individuals’ cognitive functioning. In addition, this study extends the application of COR theory to the field of digital hoarding research.

Third, drawing on the Individual-Environment Interaction theory, this study identifies the boundary conditions of digital hoarding-induced cognitive failures, specifically the moderating role of mindfulness. The findings indicate that individuals with different levels of mindfulness show significant differences in their emotional responses and cognitive performance when faced with large amounts of unprocessed digital information. This finding provides theoretical support for the development of scientifically effective psychological prevention strategies and the design of personalized interventions in the future.

### 6.2 Practical implications

This study highlights the critical role of mind-oriented digital wellness initiatives in helping young users navigate the challenges of the digital society. Accordingly, students should engage in mindfulness training through diverse and adaptable practices to strengthen their mindfulness skills. For example, digital mindfulness, which combines traditional mindfulness principles with advanced technologies such as mobile apps, wearable devices, and virtual reality, makes mindfulness practice more accessible and personalized (Yosep et al., 2024). A recent study suggests that digital mindfulness-based interventions can significantly reduce repetitive negative thinking (Vargas-Nieto et al., 2024). In addition, loving-kindness meditation, which emphasizes the cultivation of love and kindness toward oneself and others, could be seen as a promising alternative for increasing positive psychological capital and resilience (Chiou et al., 2021; Liu et al., 2021). Studies by Liu et al. (2023) have shown that short video applications of LKM can help college students effectively cope with psychological pressure and reduce academic anxiety. Therefore, we encourage students to systematically develop mindfulness through a variety of innovative methods to learn basic practices, including Loving-Kindness Meditation, Mindful Breathing and Body Scan Meditation. We believe that incorporating mindfulness into daily life can help young digital hoarders navigate the information overload of the digital age more calmly and achieve a rational balance between collecting and using information.

## 7 Limitations and future directions

While this study has produced some strong findings, it also has certain limitations that need to be addressed in future research. First, this study utilized a cross-sectional design, which limits the ability to draw causal inferences. Future research should consider using a longitudinal design to further explore the causal relationship between digital hoarding and cognitive failures. Second, given that college students are a high-risk group for digital hoarding, the sample in this study primarily consisted of college students. Future research could expand the participant pool to include different age groups to further test the existing findings. Additionally, individuals from different countries and cultural backgrounds may exhibit variations in digital hoarding behavior, so future studies could conduct cross-cultural comparisons to examine the potential moderating role of cultural differences in the impact of digital hoarding on cognition and emotions. Finally, this study relied on self-report questionnaires, which may introduce self-report bias. Future research could explore more reliable measures. For example, techniques such as Event-Related Potentials (ERP) or Functional Magnetic Resonance Imaging (fMRI) could be used to examine the brain function of digital hoarders from a neurological perspective.

## 8 Conclusion

This study identifies the relationship between digital hoarding and cognitive failures in college students and the underlying mechanisms. The main findings are: (1) Digital hoarding positively predicts cognitive failures. (2) Fatigue mediates the relationship between digital hoarding and cognitive failures. (3) The direct and indirect relationships between digital hoarding and cognitive failures, mediated by fatigue, will vary from person to person. Specifically, mindfulness as an individual trait not only reduces the impact of digital hoarding on cognitive failures but also moderates the mediation process of “digital hoarding – fatigue – cognitive failures.” The findings have important theoretical and practical implications for advancing research on the relationship between digital hoarding and individual cognitive and behavioral maladjustment. They also provide valuable guidance for college students in promoting their mental well-being and optimizing digital information management.

## Data Availability

The raw data supporting the conclusions of this article will be made available by the authors, without undue reservation.

## Footnotes

^1^ www.wjx.cn
